# Patients’ attitudes and expectations toward a digital inpatient-like psychotherapy concept: a qualitative interview study

**DOI:** 10.1186/s12888-026-08390-6

**Published:** 2026-07-17

**Authors:** Lucy Ann Gresser, Tania Lalgi, Patrick Jonas Wollenberg, Alexander Diel, Christoph Jansen, Rebekka Robitzsch, Martin Teufel, Alexander Bäuerle, Anita Robitzsch

**Affiliations:** 1 Clinic for Psychosomatic Medicine and Psychotherapy, LVR-University Hospital Essen, University of Duisburg-Essen, Virchowstraße 174, 45147 Essen, Germany; 2 Center for Translational Neuro- and Behavioral Sciences (C-TNBS), University of Duisburg-Essen, Hufelandstraße 55,, 45147 Essen, Germany

**Keywords:** E-mental health, eHealth, Psychiatric inpatient care, Digital intervention, Blended psychotherapy, Mental health care

## Abstract

**Background:**

In recent years, digital mental health interventions have become increasingly important due to the rising demand for psychotherapy and the shortage of resources. Digital inpatient-like psychotherapy (DIPT) is a therapeutic concept that allows patients to receive inpatient-like psychotherapy in a digital environment, encompassing multiple dimensions of psychotherapy. It represents a comprehensive and intensive form of psychotherapeutic treatment.

**Objective:**

This qualitative interview study sheds light on patients’ attitudes and expectations toward digital inpatient-like psychotherapy in terms of acceptance, perceived benefits and barriers, as well as suggestions for initial implementation steps.

**Methods:**

Semi-structured interviews were conducted with 20 patients receiving day-patient or inpatient psychotherapy in a university hospital. Iterative thematic analysis and inductive coding were conducted by three independent researchers following Braun and Clarke’s approach to thematic analysis. Three overarching themes were identified, along with multiple subsidiary themes and subthemes.

**Results:**

Interviewees included individuals with various mental health disorders. The analysis of the interviews revealed three overarching themes: (1) requirements for effective implementation of DIPT, (2) relational dynamics within the interpersonal domain of DIPT, and 3) relief associated with engagement in DIPT. These overarching themes comprised six themes. The overall attitude and acceptance toward digital inpatient-like psychotherapy was predominantly positive, with interviewees recognizing numerous benefits including location independency, time flexibility, and increased openness toward personal mental health issues. However, potential barriers such as lack of personal prioritization, tendencies for social withdrawal or insufficient relationship building must be addressed and considered.

**Conclusion:**

The findings provide valuable insights into digital mental health interventions and could assist in the development of feasible, patient-centered digital inpatient-like psychotherapy concepts. It is essential to consider patients’ appreciation for personal interaction, which could be addressed through the approach of a hybrid treatment.

**Supplementary Information:**

The online version contains supplementary material available at 10.1186/s12888-026-08390-6.

## Introduction

Poor mental health imposes substantial social and economic burdens, affecting individuals’ education, employment and physical well-being [1]. The prevalence of mental health disorders has almost doubled since 1990 [2]. According to estimates from the Global Burden of Disease study, the prevalence of major depressive disorders and anxiety disorders has increased by 27.6% and 25,6%, respectively, since 2020 [3]. In Germany, the one-year prevalence of mental disorders is approximately 27.7% [4]. As the prevalence of these disorders continues to rise, the demand for psychotherapy has increased accordingly, leading to longer waiting times to begin psychotherapy [5, 6].

Only a little more than half of the patients receive psychotherapy within a six-month waiting period [7]. Moreover, mental disorders incur significant costs, both due to the need for a functioning health care system and the financial support required for work incapacity [8], with inpatient care being particularly impacted [9]. Inpatient psychotherapy plays a crucial role in mental health care, as it provides more intensive and multimodal treatment compared to outpatient care, and is often required due to severe symptoms [10, 11]. However, there is a critical need for improvements in cross-sectoral and interdisciplinary care models [12]. In addition, treatment resources are constrained by staffing shortages and limited bed availability [13, 14]. Moreover, stigmatization, particularly in inpatient care, represents a significant obstacle for patients [11]. In conclusion, many individuals with mental health conditions are unable to access appropriate treatment due to the unavailability of services, decreased capacities or inaccessibility [15].

A growing transition toward digital health interventions is emerging, as these health services play an increasingly vital role in the health-care system [16]. The implementation of digital interventions holds significant potential to alleviate strain on the health care system by conserving scarce resources – such as through cost reductions and decreased inpatient stays [17, 18]. Digital psychotherapeutic approaches can provide highly accessible, multimodal and multiprofessional treatment [19–21]. Numerous randomized controlled trials have been conducted to assess the efficacy and feasibility of digital mental health interventions [22–25] with results indicating significantly greater effectiveness compared to control groups on waiting lists [26]. Additionally, a substantial improvement in the quality of life has been observed in patients with depression or anxiety [25, 27, 28]. However, patients’ responses to digital interventions can vary. While some studies show high acceptance [29–31], other studies report relatively low completion rates [32–34].

Digital inpatient-like psychotherapy (DIPT) is a theoretical therapeutic concept that allows patients to receive inpatient-like psychotherapy in a digital environment. In Germany, inpatient or day-patient psychotherapy typically involves a more comprehensive and intensive form of psychotherapeutic treatment. The concept of DIPT encompasses digital diagnostics, structured video sessions, home-monitoring tools, and video-based psychotherapeutic procedures such as individual and group treatments, interactive exercises, psychoeducation, and mindfulness-based interventions. Investigating the predictors of acceptance, as well as the needs and expectations of patients who could benefit from a DIPT concept, provides valuable insights into their perspectives. This understanding facilitates the development of user-centered therapies that align with individual needs, thereby enhancing the effective management of patients’ mental health conditions. Furthermore, an adapted digital therapy approach could lead to higher acceptance and increased utilization of digital applications [35–37]. Since DIPT is a novel concept that has not yet been formally established, there is currently one peer-reviewed research specifically addressing it [38]. Existing studies primarily focus on complementing inpatient care with digital elements [39–41]. Moreover, numerous studies focus on various aspects of digital psychotherapy, such as internet-based emotion regulation [23, 42] or app-based cognitive behavioral therapy [24, 43], typically addressing a single digital therapeutic component. Unlike traditional approaches that may focus on single therapeutic components, the concept of a DIPT provides a holistic framework that addresses multiple dimensions of psychotherapy simultaneously and creates a digital environment to deliver inpatient-like treatment. This concept was modeled after the scope and intensity of inpatient and day-patient psychotherapy, which provides a broader spectrum of therapeutic opportunities.

This study aims to shed light on patients’ attitudes and expectations toward DIPT by applying a qualitative research approach. Since DIPT is a self-developed concept that still requires further development, this qualitative study aims to explore, on a theoretical level, the conditions under which such a concept could be successful. Specifically, it seeks to identify which components may be necessary for effective implementation, in which contexts DIPT could be feasible, and in which direction the concept might evolve. Particular attention is paid to the need for flexibility, as DIPT may require adaptation to different contexts, patient groups, and therapeutic settings. The specific goals are to assess the overall perception of such a concept and to identify its perceived advantages, challenges, and barriers, particularly concerning its implementation. By providing a nuanced understanding that goes beyond quantitative measures, the findings will not only advance implementation research but also inform the development of health policy recommendations for the integration of such treatment approaches into mental health care.

## Methods

### Study design

This study is based on a qualitative framework using semi-structured interviews to explore key aspects of patients’ attitudes and expectations. The semi-structured interview framework can be found in Appendix A (Table A1). The study was conducted in accordance with the Consolidated criteria for Reporting Qualitative Research (COREQ) guidelines [44]. The COREQ-Checklist can be found in Appendix A (Table A2). Prior to the start of the study, the Ethics Committee of the Medical Faculty of the University of Duisburg-Essen approved the study (23-11642-BO). Interviews were conducted from 3/22/2024 to 5/21/2024.

### Interviewees and researchers

Twenty patients (10 male, 10 female) between 22 and 65 years old (*Mean* 40.35; *SD* 14.37) affected by various mental health disorders including depression, somatoform disease, post-traumatic stress disorder, anxiety disorder, and anorexia nervosa participated in the study. Inclusion criteria included: (1) patients with a mental health disorder receiving day-patient or inpatient care; (2) who are at least 18 years old; and (3) are fluent in German.

The research team conducting this qualitative study has a background in clinical psychology, psychosomatic medicine and psychotherapy with individual (qualitative-) research experience (full-degree professor, post-doctoral researchers, and Ph.D. candidates).

### Recruitment

Recruitment was done through advertisements displayed in the LVR-University Hospital Essen, Clinic for Psychosomatic Medicine and Psychotherapy, or through personal introductions to eligible patients inquiring about their interest in participating. DIPT has not yet been implemented in this facility. Nineteen out of twenty interviewees (95%) were recruited through personal introductions, while one interviewee contacted the research team after seeing the flyer and expressed willingness to participate. Every patient who started the interview completed it.

### Semi-structured interview

Through a qualitative approach using semi-structured interviews, comprehensive insights into patients’ attitudes and expectations can be obtained. This approach allows interviewees to freely answer open-ended questions, capturing their initial thoughts and emotions. A semi-structured interview gives the interviewer flexibility to explore beyond the predefined questionnaire, focusing on new and interesting aspects raised by the interviewee [45]. The research team focused particularly on personal motives, factors influencing attitudes, and the proper implementation of DIPT from the patients’ unique perspective.

The interview began with sociodemographic questions (e.g., gender, age) and continued with inquiries about the technical environment and usage of technologies (e.g., handling, possession, stable internet connection, daily usage, use of digital media related to mental health problems). Interviewees were then asked about their familiarity with the concept of DIPT and how they would define it, if familiar. Afterwards, all interviewees received a standardized definition and explanation of the concept of DIPT and had the opportunity to ask questions for further clarification. The goal was to provide interviewees with an overall understanding of the concept without imposing overly restrictive parameters that might constrain their own reflections and ideas.

Following this, we conducted semi-structured interviews using an interview guide as a framework with open-ended questions, allowing interviewees to respond freely while enabling the interviewer to ask follow-up and probing questions as needed. The questionnaire covered four main aspects: the facets of attitude toward DIPT, positive aspects, constraints and obstacles, and implementation requirements. Some questions (questions: 5, 6, 8, 13, 14, 15) focused on the direct determinants of the Unified Theory of Acceptance and Use of Technology [36, 46]. The interview questions and the standardized definition of DIPT are provided in Appendix A (Table A1 and A3).

### Data collection

Written informed consent was obtained after providing verbal and written descriptions of the study. All interviews took place on-site and were audio recorded. The interviews were conducted with consideration for saturation, which was achieved after 20 interviewees. Data collection ceased once no additional essential information was identified from further interviewees. This decision was made through the regular transcription and review of the completed interviews, as well as the non-systematic notes recorded for each interview [47]. The auditory data will be stored on a password-protected device and retained for 10 years.

### Quality control

To ensure the quality and validity of the study, the COREQ guidelines were followed for the interview questions. To achieve high reliability, two independent researchers coded the data. Additionally, a cross-coded procedure was conducted by another independent researcher. This method, based on Kuckartz [48], resulted in high intercoder concordance for themes and subthemes, as previous divergences were eliminated. As an iterative process, researchers regularly met to address discrepancies and share research and analysis results from the transcripts, forming their understanding and perception of the presented data [49].

### Data analysis

**Fig. 1:**
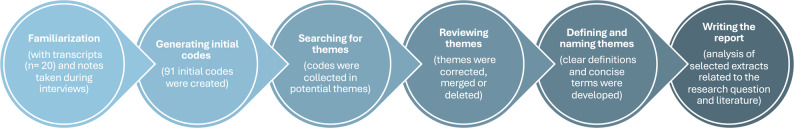
Flowchart of the data analysis process based on Braun and Clarke’s thematic analysis approach [51]

Twenty pseudonymized transcripts were collected. Interview duration varied from 28 to 61 min (*mean* 39.25 min, *SD* 9.44 min). To ensure accuracy, audio files were transcribed verbatim into text files using the transcription software f4x [50] and were proofread to ensure complete alignment between spoken and written words. Audio data was securely stored. The data was analyzed, structured, and coded using the MAXQDA software (version 2024). Thematic analysis was conducted using Braun and Clark’s (2006) six-step iterative process (see Fig. 1). Initially, two independent researchers explored and analyzed 50% of the transcripts, and a third independent researcher explored 25% of the transcripts. Subsequently, initial codes and themes were collaboratively generated. All data were then analyzed in detail, and the codes and themes were reviewed and verified. Finally, the themes were organized into overarching themes, themes and subthemes to provide a clearer structure and overview of the findings. This inductive approach facilitated the identification of themes and codes derived from patients’ perspectives, prioritizing their essential concerns and discussions, instead of confining their thoughts to pre-established themes [51]. This method enables a deeper understanding of individual and diverse perspectives, helping to better grasp the acceptance, expectations, and needs of a DIPT.

## Results

### Overview

The interview opened with general questions about the daily use of digital devices. Every interviewee owns a phone and laptop, or computer and 95% of the interviewees (19 out of 20) possess a stable internet connection. Sociodemographic, diagnosis-related and use of digital media characteristics of the interviewees are shown in Table 1.

**Table 1:** Sociodemographic, diagnosis-related and use of digital media characteristics

Characteristic	Values	
	*N*	(%)
Age (in years)		
20–29	7	(35)
30–39	4	(20)
40–49	3	(15)
50–59 60–69	3 3	(15) (15)
Reason of treatment		
Depression	5	(25)
Somatoform disorder	5	(25)
Post-traumatic stress disorder	4	(20)
Anorexia nervosa Anxiety disorder	5 1	(25) (5)
Media competence in everyday life		
Well	14	(70)
Ok Bad	6 0	(30) (0)
Use of digital technologies		
Daily	19	(95)
A few times a week Once a week Rarely Never	1 0 0 0	(5) (0) (0) (0)
Use of digital media (e.g., health apps, research) according to mental health problems		
Yes	10	(50)
No	10	(50)

Note: *N* = 20

### Overarching themes

**Fig. 2:**
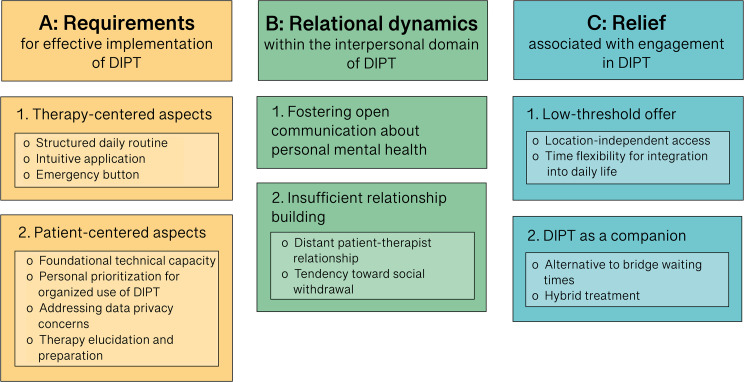
Graphical representation of overarching themes **A, B** and **C**, themes and subthemes generated from the interview transcripts

Three overarching themes construed inductively from the interview transcripts: (1) requirements for effective implementation of DIPT, (2) relational dynamics within the interpersonal domain of DIPT, and 3) relief associated with engagement in DIPT. From these overarching themes, a total of six themes were identified, each accompanied by corresponding subthemes. A graphic display of the overarching themes, themes and subthemes can be seen in Fig. 2. Table A5 provides further quotes for the themes and subthemes.

## Overarching theme A: Requirements for effective implementation of DIPT

### Theme 1: Therapy-centered aspects

This theme explores the key characteristics that interviewees believe DIPT should encompass. The following subthemes were frequently mentioned and were considered essential for facilitating an accessible approach to DIPT.

#### Subtheme 1.1: Structured daily routine

One interviewee stated, *“Everyone needs a solid structure in therapy” (Interviewee 2)*. Overall, the majority of the interviewees expressed the need for a structured therapy plan. A structured routine enables them to take time for therapy, complete daily tasks, and find consistency in a DIPT (see quote [1]). It facilitates the entire therapy process and provides personal direction (see quote [2]). While one interviewee indicated to *“…put therapy modules into individual time slots” (13. Interviewee)*, another said, *“Putting the therapy modules together myself would make me feel overwhelmed” (15. Interviewee).*

Interviewees recommended various digital therapy modules they would like to see included in a structured DIPT. Therapy modules, along with the number of interviewees who mentioned each, are presented in Table 2.

**Table 2:** Preferred digital therapy modules

Characteristic	Values	
	*N*	(%)
One-on-one therapy Psychoeducation Group therapy	14 13 8	(70) (65) (40)
Skills	7	(35)
Sports therapy	6	(30)
Art therapy Space for notes/ diary Mindfulness/ time for reflection	6 6 5	(30) (30) (25)

Note: *N* = 20

#### Subtheme 1.2: Intuitive application

The interviewees emphasized the significance of a *“…user-friendly*,* intuitive*,* and self-explanatory…”* therapy interface *(15. Interviewee)*, and as Interviewee 6 stated, *“It would be really important to have simplified access”.* The therapy platform should not be fragmented into different systems, portals, and databases (see quote [3]). Instead, it should have a well-organized interface that helps users easily find what they are looking for (see quote [4]). Without this simplification, interviewees would *“lose interest”* in participating in DIPT *(7. Interviewee)*.

#### Subtheme 1.3: Emergency button

According to half of the interviewees, an *“emergency button” (19. Interviewee)* was seen as a crucial feature of DIPT. The availability of a contact person in emergencies, such as those involving physical symptoms, was deemed essential (see quote [5]). As Interviewee 3 noted, *“…maybe that you could press a button and ideally be connected directly. This could lower the threshold*,* as you wouldn’t have to manually enter a phone number”.* The use of a digital device, which is likely accessible in various locations because of its easy entrainment, could further support this low-threshold approach to reaching out for help (see quote [6]).

### Theme 2: Patient-centered aspects

This theme focuses on the prerequisites patients need to bring and the support they may require to successfully participate in DIPT. Interviewees highlighted the importance of foundational technical capacity and personal commitment to prioritizing DIPT. Furthermore, certain barriers, such as data privacy or a general lack of knowledge about the therapy concept, were identified. These issues could be addressed directly, thereby maximizing the benefits and efficacy of the intervention for patients while also empowering them to overcome personal challenges.

#### Subtheme 2.1: Foundational technical capacity

Three main aspects were deemed important for the interviewees regarding technical factors. First, patients need a basic level of technological literacy. As one interviewee remarked, *“This therapy would probably not work for everyone*,* but for most. Of course*,* people need to have some technical understanding” (12. Interviewee).* If patients’ technical skills are insufficient for the practical use of DIPT, they may *“…lose the motivation to keep engaging” (7. Interviewee).* Second, interviewees recognized possible technical obstacles, such as unstable internet connections or software issues, that could lead to diminished therapy quality (see quote [7]). Technical problems could cause interruptions in therapy that *“…would be disturbing and frustrating” (6. Interviewee).* A larger part of the interviewees noted the importance of having the right equipment, such as digital devices with sufficient power, as well as a webcam, microphone, and headphones (see quote [8]). One interviewee recommended that DIPT *“…should be available on both a web-based and an app-based platform” (13. Interviewee).* Third, about half of the interviewees would prefer an introduction into the technical use of DIPT to help them in their technical understanding and increase their comfort while participating in DIPT (see quote [9]). Some stated, *“I am technically skilled; a short instructional video would be completely fine for me“ (12. Interviewee)*, while others imagined additional personal and individualized assistance *“for those who have more questions or need further explanation” (13. Interviewee).*

#### Subtheme 2.2: Personal prioritization for organized use of DIPT

*"There must be a tendency toward self-organization (…). I could imagine that some patients (…) may lack motivation to pull themselves together and take matters into their own hands” (Interviewee 12).* There might not be enough inducement, depending on the individual’s daily condition (see quote [10]) and *“…there would be a higher risk of getting distracted” (14. Interviewee).* Independent, self-disciplined participation in DIPT may require a higher expenditure of energy, that some patients may not have, potentially leading to procrastination, as *“…it is easy to just put the phone aside – out of sight*,* out of mind” (1. Interviewee)*. Some interviewees voiced their concerns about being distracted by their familiar environment and household tasks that need to be completed (see quote [11]). To counteract low self-discipline and motivation, a number of interviewees suggested a notification system that reminds them to engage in therapy. As Interviewee 13 mentioned, *“The app could send notifications reminding users to take time for therapy modules. I think that could be very helpful".*

#### Subtheme 2.3: Addressing data privacy concerns

More than half of the interviewees outlined the importance of adequate data protection and clear communication regarding the privacy policies of the program used for DIPT. As one interviewee stated, *“Data protection must be guaranteed well*,* especially because sensitive data is involved” (3. Interviewee).* Several interviewees perceived a risk that sensitive data could be easily stored and accessed. In addition, there was a concern that stolen data could be misused (see quote [12]). Although data privacy was a recurring topic, the main part indicated that they had no concerns about data privacy when using DIPT (see Appendix A, Table A4). It remains unclear whether this concern stems from limited awareness of potential risks or confidence in the adequacy of existing data privacy measures.

#### Subtheme 2.4: Therapy elucidation and preparation

Several interviewees indicated that becoming familiar with the concept of DIPT beforehand would be beneficial (see quote [13]). Being informed about the structure and therapy process before commencing therapy would provide a greater sense of security (see quote [14]). Furthermore, *“It’s important that the technical requirements of the program are clearly outlined so that the patients can say: ‘Yeah*,* my computer is still good enough!’ (…) These things need to be clarified in advance” (14. Interviewee).*

## Overarching theme B: Relational dynamics within the interpersonal domain of DIPT

### Theme 1: Fostering open communication about personal mental health issues

Over half of the interviewees felt that DIPT could create a personal *“safe place” (4. Interviewee)*, as therapy occurs in a familiar and comfortable environment. This sense of safety could lead to increased openness toward personal mental health issues, as Interviewee 1 said, *“If I feel comfortable and safe in the moment (…)*,* I could imagine talking more openly about certain topics.”* Particularly, patients with social phobia could benefit from DIPT as it offers a lower threshold for initial entry into therapy, as *“…it becomes easier to connect with others” (10. Interviewee).* One interviewee compared DIPT with social media, stating, *“A lot of people have fewer inhibitions about talking about their own issues because they are talking to a camera instead of a person who is actually sitting in front of them” (19. Interviewee).* The anonymity provided by DIPT makes participants feel more *“…comfortable when they don’t have to show themselves in person” (2. Interviewee).*

### Theme 2: Insufficient relationship building

Although the overall attitude toward DIPT was very positive (see Appendix A, Table A4), some factors were mentioned that could negatively influence relationships within the DIPT. This theme addresses various aspects that could make it challenging to build close relationships with healthcare workers and fellow patients through DIPT.

#### Subtheme 2.1: Distant patient-therapist relationship

Most interviewees identified the risk of a more distanced patient-therapist relationship as a potential disadvantage. Some interviewees mentioned the missing personal interaction and the need for solidarity during therapy (see quotes [15] and [16]). This barrier was described as a *“wall”*, that could make it difficult for the patients to speak unrestricted (see quote [17]). As Interviewee 9 expressed, *“It feels like there is a wall in front of you*,* and it’s just a picture talking to you. That’s why physical closeness is important.”* On-site communication is *“representing an entirely different dimension” (14. Interviewee)* compared to digital communication. Moreover, the majority of the interviewees identified the lack of nonverbal communication as a potential constraint (see quote [18]). One interviewee suggested, *“More communication happens through gestures*,* facial expressions*,* and body language than through the spoken word. I think therapists pick up less*,* and it doesn’t come across fully” (16. Interviewee).* Furthermore, a lower concentration span, triggered by looking at a screen, could make it difficult to fully engage in DIPT interventions (see quotes [19] and [20]).

#### Subtheme 2.2: Tendency toward social withdrawal

One interviewee remarked, *“Digitalization can be a blessing and a curse at once*,* because some people may lose themselves in digital media and isolate themselves from their outside world” (3. Interviewee).* Some interviewees feared that DIPT might cause them to isolate themselves further due to their mental illness. As Interviewee 18 pointed out, *“You completely isolate yourself*,* especially when dealing with trauma. (…) Initially*,* it may be easier*,* but it won’t get you out of isolation like inpatient therapy does.”* It was suggested that patients might tend to stop DIPT when sensing personal overwhelm and could easily quit therapy, thus not receiving the help they need (see quote [21]). Furthermore, Interviewee 13 assumed that patients might *“…comfortably just pick a few modules and then maybe not finish them because their mental illness affects their ability to stick to a plan.”*

## Overarching theme C: Relief associated with engagement in DIPT

### Theme 1: Low-threshold offer

This theme addresses aspects of DIPT that facilitate its use by enabling patients to make the most out of the DIPT. Many benefits were mentioned and as Interviewee 14 stated, that a DIPT *“…would allow you to better detach from time and space.”*

#### Subtheme 1.1: Location-independent access

Every interviewee highlighted the advantage of location-independent access to DIPT. This independence provides numerous individual benefits. Firstly, regardless of where patients are, they can access DIPT (see quote [22]). Moreover, patients with physical and mental restrictions can engage in therapy (see quotes [23] and [24]). Being free from the constraints of location reduces stress before therapy sessions begin, as Interviewee 17 stated, *“It would be a relief not to have to travel to therapy.”* Furthermore, *“…the time and energy saved can be invested in the actual therapy” (8. Interviewee).* Moreover, the location independence of DIPT enables it to reach individuals living in remote or underserved regions (see quote [25].

#### Subtheme 1.2: Time flexibility for integration into daily life

A larger part of the interviewees mentioned time flexibility as a significant positive aspect of DIPT. Interviewee 2 noted, *“Since there are no opening times*,* I can plan individually.”* Several interviewees indicated that they would incorporate DIPT into their daily routines, keeping it flexible and dependent on their current state, as *“…you could do mindfulness exercises or other therapy modules when you need them the most” (12. Interviewee).* Furthermore, therapy modules can be completed within an individual time span, as Interviewee 5 said, *“I can do them at my own pace.”* Unlike on-site therapy, DIPT could not require a rigid schedule, providing more personal freedom in arranging therapy sessions (see quote [26]).

### Theme 2: DIPT as a companion

This theme explores the role of DIPT as a daily companion for patients, enabling them to receive continual therapy on a daily basis.

#### Subtheme 2.1: Alternative to bridge waiting times

Due to shortage of therapy and long waiting periods, DIPT could serve as an excellent alternative for providing faster assistance to patients, as it *“…could help people more quickly” (8. Interviewee).* One interviewee remarked, *“It can only get better because many patients who are dealing with their mental illness are waiting for available psychotherapy” (5. Interviewee).* By utilizing DIPT, *“…eventually*,* more therapy slots could be offered to more people” (19. Interviewee).*

#### Subtheme 2.2: Hybrid treatment

Most of the interviewees concluded that a combination of on-site therapy and DIPT would offer an *“enriched”* therapy experience (see quote [27]). Various approaches for therapy combinations were raised. DIPT could be offered before on-site psychotherapy, as it can *“…provide excellent first aid” (3. Interviewee)* and is *“…a good basis to start with” (1. Interviewee).* Another possibility mentioned is the simultaneous use of both therapy formats, combining on-site and digital appointments (see quote [28]). Especially therapy modules that were seen as difficult to implement in DIPT could be offered on-site and a certain amount of on-site therapy could help patients connect with each other (see quote [29]). Furthermore, DIPT could be offered after on-site psychotherapy. A digital follow-up therapy was seen as an excellent concept, especially when contact with the same people as before is being maintained (see quote [30]).

## Discussion

This qualitative study not only offers a nuanced understanding of patients’ acceptance toward a DIPT concept, but it also identifies critical individual factors essential to ensuring its successful and effective implementation. By incorporating patients’ insights, the identified aspects can be integrated into a DIPT concept as an initial step of the participatory design method [52]. Given the high demand for psychotherapy, a well-developed and tailored digital psychotherapy intervention holds considerable promise.

This study identified several key considerations for the implementation of a DIPT concept. A structured therapeutic framework is required, delivered via an intuitive digital platform accessible on both web and mobile devices. The inclusion of an emergency button was deemed essential to ensure immediate access to therapists when needed. In addition, a notification system could enhance patients’ engagement and promote higher prioritization of therapeutic activities. Technical challenges should be mitigated through technical assistance and support. Lastly, the provision of a diverse range of therapy modules tailored to individual needs is recommended to accommodate varying therapeutic requirements.

Interviewees demonstrated a high acceptance toward DIPT, consistent with previous studies assessing digital health interventions [31, 53]. Despite high acceptance and openness toward DIPT, only a few interviewees could imagine it to be a replacement for on-site inpatient therapy. Some patients felt that DIPT would not meet their needs due to insufficient relationship building throughout the therapeutic process. This substantial obstacle is reflected in other studies as well [19, 54]. Additionally, certain therapy modules, such as exercise or art therapy, seemed difficult to realize digitally. Consequently, many interviewees proposed the idea of a hybrid treatment combining on-site and digital psychotherapy. These findings align with previous research, where blended therapy – the combination of face-to-face therapy with digital elements – is seen as a potential model for more efficient psychotherapy [55–57].

Frequently mentioned benefits included location independence, time flexibility, the ability to bridge waiting periods, and increased openness in communication about personal mental health. These aspects would facilitate the adoption of DIPT and extend its reach and accessibility. Location independency and time flexibility were particularly valued, consistent with findings from other studies [58, 59]. Through this digital concept the interdisciplinary therapeutic approach could be extended across locations, enabling health care workers from different facilities to collaborate. This could counteract staff shortages and improve coordination between healthcare workers [60–62].

DIPT was perceived as an excellent way to bridge the gap between patients on the waiting lists and the start of actual psychotherapy [63]. A long treatment gap can create frustration in patients and lead to therapy discontinuation, which may result in aggravated symptoms [64, 65]. From the interviewees’ perspective, DIPT could help avoid this. Conversely, some studies show no remarkable difference in outcomes between digital interventions during waiting times and control groups who merely waited or received self-help literature [35]. One must consider that the effectiveness of digital interventions depends on their quality [37]. Moreover, it cannot be generally concluded that a DIPT concept can be implemented with fewer healthcare workers; it still requires sufficient personnel to maintain high-quality therapy. However, such concepts can use location independent resources (e.g., experts).

Increased openness, enabling patients to share their problems more freely, was regarded as an immense gamechanger, as DIPT could allow patients to confront their issues more directly, fostering a sense of safety and reducing feelings of being overwhelmed. These positive emotional responses are attributed to the supportive domestic environment in which patients receive DIPT. A qualitative study from the perspective of therapists highlighted relational connectedness, using terms such as: “*open*”, “*personal*” or “*trusting*” [66].

Conversely, potential obstacles include insufficient relationship building, technical problems, usability challenges, and privacy concerns. These obstacles are comparable to the perspective of health care professionals, who identified restricted technical capability, struggling with concentration, and data safety issues as possible barriers [53, 59]. Some of the mentioned obstacles were deemed conquerable through measures such as introductory seminars on using devices and digital platforms. Introductory seminars and explanations are crucial for personal application and may lead to higher adherence. In addition, many patients depend on the assistance of healthcare professionals for accessibility to web-based therapies [59].

Therapy-centered and patient-centered aspects must be fulfilled to enable higher acceptance and support within DIPT. DIPT concepts should provide daily structure for patients, include an “emergency button”, and its digital platform should be as intuitive as possible. Participants must possess basic technical skills, sufficient hard- and software, and a prioritized organized use. One may argue that people with a remarkably higher number of mental diagnoses and symptoms may show greater inactivity toward online psychotherapy [42]. Reasons for not participating in digital psychotherapy could be for instance a high level of depression [33]. But a discontinuity in treatment is associated with an unfavorable prognosis [67]. Since personal prioritization is influenced by a range of intrinsic and extrinsic factors, it may not be fully enhanced by the structure of DIPT or by personal support alone. A continued emphasis on individual prioritization remains essential.

Furthermore, the needs and demands for DIPT were seen as unique. Individual psychotherapy options modified to patients’ symptoms, diseases and personal preferences are deemed essential for successful DIPT. Adaptive interventions tailored to the person, moment and context in daily life are considered primary and effective digital forms of care [37, 67]. Nonetheless, the empirical evidence for personalization in digital mental health interventions is deficient and equivocal, requiring further exploration [68].

While this study provides a deeper understanding and a nuanced patient’s perspective on DIPT, certain limitations were drawn and need to be addressed in further research. Firstly, while a qualitative paradigm contributes in-depth information that can be applied to other settings, it lacks generalizability to a larger community [69]. Furthermore, patient recruitment took place through a specialized hospital for psychosomatic medicine and psychotherapy, which may offer a level of psychotherapy not widely available in other countries. Consequently, the concept of DIPT may need to be adjusted in settings with less frequent access to psychotherapy, depending on local infrastructure and available resources. Therefore, the adaptation of DIPT in other countries should be tested to explore its transferability. Additionally, there is also a risk of selection bias, as interviewees may have exhibited a general openness toward the topic. Potential interviewees with little interest might have declined to participate in the study. However, during the recruitment process, the researcher was unaware of the interviewees’ level of interest. Moreover, by presenting a standardized definition of DIPT necessary to ensure uniform knowledge some aspects may have been adopted by interviewees without fully reflecting their initial, unprompted views and thoughts.

## Conclusion

Overall, there was a high level of acceptance for the concept of DIPT. Interviewees expressed that DIPT could foster openness toward personal mental health issues, as it provides a safe place within a familiar environment. However, limitations on relationship building, should be considered and addressed. Furthermore, key steps to implementation have been highlighted and should guide future development. Interviewees suggested the feasibility of a hybrid treatment model that combines digital psychotherapy with on-site psychotherapy. In conclusion, a digital inpatient-like therapy concept could be leveraged to consolidate resources and expertise and thus improve care.

## Supplementary Information

Below is the link to the electronic supplementary material.

**Supplementary Material:** Appendix A

## Data Availability

Data that supports the findings of this study are available from the corresponding author upon reasonable request.

## Abbreviations

COREQ: Consolidated Criteria for Reporting Qualitative Research
DIPT: Digital Inpatient-like Psychotherapy
