# Supplementary material for: Patients’ attitudes and expectations toward a digital inpatient-like psychotherapy concept: a qualitative interview study
Source: BMC Psychiatry. 2026 Jul 17;26:542. doi: 10.1186/s12888-026-08390-6 (PMC13383404; doi:10.1186/s12888-026-08390-6)
Supplement: Supplementary file 1 — Supplementary Material: Appendix A [file 12888_2026_8390_MOESM1_ESM.docx]

**Appendix A**

**Table A1**

*Semi-structured interview:* ***Translated (originally in German) for publication purpose only***

| Segments | Questions |
| --- | --- |
| **Facets of attitudes toward digitalized therapy** | 1. What are your general impressions and experiences with the use of digital technologies and media in the healthcare system or in the context of your medical care *(e.g., computers, laptops, mobile devices, smartphones, internet, social media, communication platforms, artificial intelligence)?* 2. What are your thoughts on the implementation of digitalization in medicine? 3. What is your opinion on the implementation of DIPT in our clinic? 4. How do you feel about receiving DIPT instead of on-site therapy? 5. Can you imagine that DIPT could address your individual mental health issues as effectively as conventional therapy? 6. Is your attitude toward DIPT influenced by personal experiences, stories from friends and family, or your social environment? 7. Do you feel that DIPT could have a different effect on you or feel different compared to conventional therapy?   🡪 If so, could you describe how it would feel different and what differences would stand out? |
| **Positive aspects** | 1. What advantages do you see in DIPT? 2. How would your inhibition threshold toward starting psychotherapy change if it was offered digitally? |
| **Constraints and obstacles** | 1. What disadvantages do you see in DIPT? 2. In your opinion, what obstacles might you face while participating in DIPT *(e.g., bad internet connection)*? 3. Are there any aspects that would make it difficult for you to participate in DIPT (*e.g., concerns about data privacy)?* |
| **Implementation requirements for a digitalized therapy** | 1. In your opinion, what personal requirements or circumstances must be met to make DIPT successful for you? 2. What digital elements need to be part of a holistic DIPT to effectively and specifically treat your mental health condition? 3. What elements or conditions would facilitate a better start into DIPT? 4. How would you incorporate DIPT into your daily routine? 5. How important is regular digital contact with healthcare workers through messages, calls, and video conferences to you?   🡪 Could you explain why this is important or unimportant to you?   1. Would you like to be in contact with other patients (receiving similar therapy) through chatrooms, video conferences, virtual reality…?   🡪 Why would this be important or less important to you? |

*Note.* Semi-structured interview that was conducted in the present study.

**Table A2:**

*Consolidated criteria for reporting qualitative research (COREQ; [1])*

| **Domain 1: Research team and reflexivity**  *Personal characteristics* | |
| --- | --- |
| 1. Facilitator | Lucy Ann Gresser, Patrick Jonas Wollenberg, Rebekka Robitzsch, Tania Lalgi, Alexander Bäuerle, Martin Teufel, Anita Robitzsch |
| 1. Credentials | Lucy Ann Gresser, Patrick Jonas Wollenberg, Rebekka Robitzsch, Tania Lalgi (M.Sc.), Anita Robitzsch (MD), Christoph Jansen (MD), Alexander Diel (PHD), Alexander Bäuerle (PHD), Martin Teufel (Prof; MD) |
| 1. Occupation | **Research associate:** Lucy Ann Gresser, Patrick Jonas Wollenberg, Rebekka Robitzsch, Tania Lalgi, Sina Bruder, Christoph Jansen  **Postdoctoral researcher:** Alexander Diel, Anita Robitzsch, Alexander Bäuerle  **Full-time professor:** Martin Teufel |
| 1. Gender | **Woman:** LAG, TL, RR, AR, SB  **Man:** PJW, CJ, AD, AB, MT |
| 1. Experience and training | **LAG:** PhD candidate  **PJW:** PhD candidate  **RR:** PhD candidate**,** assistant doctor for psychosomatic medicine and psychotherapy  **TL:** educational background in psychology (M.Sc.), doctoral researcher  **SB:** educational background in psychology (B.Sc.) , research assistant  **AR:** postdoctoral researcher and head of outpatient unit  **CJ:** assistant doctor for psychosomatic medicine and psychotherapy  **AD:** postdoctoral researcher  **AB:** postdoctoral researcher and head of research for psychosomatic medicine and psychotherapy  **MT:** full-time professor for psychosomatic medicine and psychotherapy |
| ***Relationship with participants*** | |
| 1. Relationship established | Participants consisted of the LVR University-Clinic of Essen for psychosomatic medicine and psychotherapy |
| 1. Participants’ knowledge of facilitator | The participants in the present study had no prior relationship established to the facilitator |
| 1. Facilitator characteristics | No other characteristics were reported about the facilitator |

| **Domain 2: Study design** | |
| --- | --- |
| ***Theoretical framework*** | |
| Methodological orientation and theory | Qualitative thematic analysis by Braun and Clark (2006) |
| ***Interviewee selection*** | |
| Sampling | Recruiting from 3/22 to 5/21/2024 |
| Method of approach | Recruiting via flyer and personal introduction |
| Sample size | *N* = 20 participants |
| Non-participation | Nonresponses from 9 individuals |
| ***Setting*** | |
| Setting of data collection | Face-to-face |
| Presence of non-participants | None |
| Description of sample | Between 22 and 65 years old (*Mean* 40.35; *SD* 14.37),  10 females, 10 males |
| ***Data collection*** | |
| Interview guide | Provided as supplemental material |
| Repeat interviews | None |
| Audio/visual recording | Audio recording |
| Field notes | Yes |
| Duration | 28 - 61 minutes (*mean* 39,25 min, *SD* 9,44 min) |
| Data saturation | Yes |
| Transcripts returned | No |

| **Domain 3: Analysis and findings** |  |
| --- | --- |
| Number of data coders | Coding by LG, TL, SB |
| Description of coding tree | Yes (in the results section) |
| Derivation of themes | (1) Requirements for effective implementation of DIPT  (2) Influence of familiar environment and digital communication on the interpersonal level  (3) Patients’ benefits from a DIPT |
| Software | MAXQDA 2024 |
| Participant checking | No |
| ***Reporting*** | |
| Quotations presented | Yes |
| Data and findings consistent | Yes |
| Clarity of major themes | Yes |
| Clarity of minor themes | Yes |

**Table A3:**

*Definition of DIPT:* ***Translated (originally in German) for publication purpose only***

| Within the context of the ongoing digitalization in healthcare, both the opportunities and the requirements for patient-oriented, comprehensive and individualized treatment of mentally ill people are increasing. The innovative concept of a digital clinic aims to enable patients and healthcare providers to discover and optimally utilize these new opportunities.  The digital clinic offers the possibility of low-threshold and flexible multimodal as well as multiprofessional treatment in a digital environment. Due to its spatial independence, it is accessible without restriction - even for external providers (experts). It is barrier-free and allows for individualized treatment intensity.  In addition to digital diagnostic, video consultations, and home-monitoring methods (e.g., wearables and mobile biofeedback), patients have the opportunity to flexibly combine therapy components, integrate them into their daily lives, and actively shape their treatment. This not only strengthens the sense of autonomy but also the responsibility for their own therapy.  Evidence-based psychotherapeutic procedures (e.g., internet-based cognitive behavioral therapy) are provided via video in sequential individual and group treatments, which are complemented by selected psychotherapy apps (e.g., with interactive exercises, videos, audios for psychoeducation, mindfulness, etc.).  The primary point of contact for all functionalities and offerings is an intuitively usable/structured clinic app. Through the use of virtual reality, the digital treatment is made experienceable for patients and providers, bringing them together.  The digital clinic, as an interface between (partial) inpatient and outpatient therapy, enables an individualized addition and further development in the sense of holistic patient care. |
| --- |

**Table A4:** DIPT

| Characteristic | Values | |
| --- | --- | --- |
|  | N | (%) |
| Familiarity with DIPT |  |  |
| Yes | 6 | (30) |
| No | 14 | (70) |
| Point of contact with digital psychotherapy (through friends, family and environment) |  |  |
| Yes | 4 | (20) |
| No | 16 | (80) |
| Openness toward DIPT |  |  |
| High | 17 | (85) |
| Partly  Low | 3  0 | (15)  (0) |
| Preferred therapy |  |  |
| Digital  On-site | 5  7 | (25)  (35) |
| Combination  Unsure | 7  1 | (35)  (5) |
| Change of inhibition of threshold* |  |  |
| Higher | 2 | (10) |
| Similar  Lower | 10  8 | (50)  (40) |
| Privacy concerns |  |  |
| Yes  No  No answer | 5  13  2 | (25)  (65)  (10) |
| Interest in digital communication with healthcare professionals |  |  |
| High | 18 | (90) |
| Low | 2 | (10) |
| Interest in digital communication with fellow patients |  |  |
| High  Low | 16  4 | (80)  (20) |

*Note*. *N* = 20.

** The reported change of inhibition of threshold through DIPT compared to
 on-site therapy suggested by the interviewees*

**Table A5**

*Quotes for subthemes****: Translated (originally in German) for publication purpose only***

| **Overarching Theme A** | |
| --- | --- |
| **Subtheme 1.1:**  **Structured daily routine** | [1] “(The DIPT needs to include) a structured daily routine so that one can complete tasks, have time for them, and maintain regularity.” (5. Interviewee)  [2] “(A daily schedule) would make it easier for me (…). I personally need something to focus on.” (4. Interviewee) |
| **Subtheme 1.2:**  **Intuitive application** | [3] “And that there’s a clear structure throughout the therapy so that I don’t have to piece things together from different systems, portals, and data repositories.” (14. Interviewee)  [4] ”(A possible barrier would be) a confusing app (…). Like, if I only reach the page after 4,000 links.” (18. Interviewee) |
| **Subtheme 1.3:**  **Emergency button** | [5] “That could actually be quite good with this digital thing, if you had a contact person you could call (…) when you notice physical symptoms or so.” (5. Interviewee)  [6] “I could imagine that digital immediate aid is feasible, since you’re more likely to have your phone with you, and maybe that would lower the threshold to call someone.” (12. Interviewee) |
| **Subtheme 2.1**  **Foundational technical capacity** | [7] “The quality of the therapy could eventually suffer, because it’s hard to ensure high quality when there are problems with the Internet connection, audio, app, or if the therapist experiences technical issues. You can’t guarantee 100% digital quality.“ (2. Interviewee)  [8] “First, you have to check what kind of device you have and whether it can do the job. Then you need a webcam, since some PCs don’t have a camera. You might also need a microphone or headphones, depending on your location. Getting the right equipment isn’t cheap.” (10. Interviewee)  [9] “I’m definitely not a technical expert. A technical introduction would probably be helpful. At the very least, it would be great to have a proper introduction or some kind of support available when facing technical problems. (…).” (17. Interviewee) |
| **Subtheme 2.2:**  **Personal prioritization for organized use of DIPT** | [10] “At home, I might not have the motivation to do the therapy on my own, depending on how I’m feeling that day.” (18. Interviewee)  [11] “Maybe I wouldn’t be able to fully commit to the therapy because I’d be distracted by my familiar home environment. I might just stick to my regular routine and only spend two and a half hours per day on therapy.” (20. Interviewee) |
| **Subtheme 2.3**  **Addressing data privacy concerns** | [12] “The issue is that digital data is very easily accessible and has to be stored somewhere. There’s always the worrying possibility that the data could be misused. Naturally that creates concern and hesitation.“ (14. Interviewee) |
| **Subtheme 2.4:**  **Therapy elucidation and preparation** | [13] “(A better introduction into the DIPT could be achieved through) first of all, a personal conversation where I receive an introductory explanation (…). What will the therapy process be like? What’s coming up for me? What do I need?“ (10. Interviewee)  [14] “I need to have a schedule. What will happen during the introduction? What will happen in the therapy sessions? What’s happening in the next 4-6 weeks? Maybe I’ll have different options. That would help me. I need a plan.” (18. Interviewee) |
| **Overarching Theme B** | |
| **Subtheme 2.1:**  **Distant patient- therapist relationship** | [15] “(In a DIPT) I wouldn’t have the certainty that I have my group and my therapists (…).” (7. Interviewee)  [16] “Somehow, I missed the personal contact. It wasn’t bad - better than I expected, but there still wasn’t a real personal connection.” (20. Interviewee)  [17] “I feel like there’s a wall. I’d have trouble sharing as openly as I would in a face-to-face setting.” (18. Interviewee)  [18] “Facial expressions and gestures, which I think are also important, might be lost in digital psychotherapy.” (6. Interviewee)  [19] “I believe it is more tiring to sit in front of a screen because your eyes get strained from staring at it. (10. Interviewee)  [20] “Looking at a screen takes too much of my concentration, and I can’t really stay engaged in a conversation for long.” (3. Interviewee) |
| **Subtheme 2.2:**  **Tendency toward social withdrawal** | [21] “Of course, you have the option to simply turn it off when it becomes too much. Some people may tend not to contact anyone anymore and, as a result, wouldn’t get the help they normally would.” (1. Interviewee) |
| **Overarching Theme C** | |
| **Subtheme 1.1: Location independent access** | [22] “Actually, it shouldn’t matter at all. If I want to do it in the car, then I can do that.” (1. Interviewee)  [23] “For example, if someone is overweight or who can’t attend therapy due to medical reasons, then doing it online would be an advantage.” (2. Interviewee)  [24] “I’ve noticed that many fellow patients isolate themselves and can’t leave the house or stay in contact. I could imagine seeking professional help to some extent would be very helpful.” (3. Interviewee)  [25] “People living farther away, in surrounding cities for example, would have a greater chance of accessing this (DIPT).” (6. Interviewee) |
| **Subtheme 1.2**  **Time flexibility for integration into daily life** | [26] “So I could do it later if I have a rough start to the day. I could design it more freely; it wouldn’t be as scheduled as it is here.” (18. Interviewee) |
| **Subtheme 2.2**  **Hybrid treatment** | [27] “I’m almost certain I wouldn’t take part in digital therapy alone. But on the other hand, I could imagine participating in a harmonious mix of digital therapy and on-site therapy – that would be enriching.” (14. Interviewee)  [28] “Maybe it (the DIPT) could be structured in stages. I don’t know, maybe one, two or three days of digital psychotherapy and the rest on-site.” (7. Interviewee)  [29] “(…) that the therapy is offered as a combination. Part of it would be digital, but other therapy forms - like ergotherapy, forest walks – would still be on-site, so that patients can connect with others.” (13. Interviewee)  [30] “I could imagine it would be much better to have a smooth transition from inpatient care into the digital world. Then you may have the same people and circumstances you already know.” (16. Interviewee) |
